# Winter syndrome: about an uncommon case report

**DOI:** 10.1186/s12905-020-00951-5

**Published:** 2020-04-21

**Authors:** Aziz Slaoui, Sarah Talib, Abdelali Kallali, Mariem Rouijel, Aziz Baydada

**Affiliations:** 1 Gynaecology-Obstetrics and Endoscopy Department, Maternity Souissi, University Hospital Center IBN SINA, University Mohammed V, Rabat, Morocco; 2 Gynaecology-Obstetrics and Endocrinology Department, Maternity Souissi, University Hospital Center IBN SINA, University Mohammed V, Rabat, Morocco

**Keywords:** Vaginal aplasia, Poly malformative syndrome, Winter syndrome

## Abstract

**Background:**

Congenital genital tract outflow obstruction may occur at different levels and with different clinical presentations. Winter syndrome was first described in 1968 as an association of renal, genital and middle ear anomalies. This syndrome is characterized by autosomal recessive transmission, unilateral or bilateral renal hypoplasia, distal vaginal atresia, and moderate to severe conductive hearing loss with malformation of the ossicles. The diagnosis is usually made when symptoms of obstruction are obvious. It presents most commonly with primary amenorrhea in a girl with a normal XX genotype, ovarian and hormone function; and cyclical abdominal pain. Ultrasound confirm the physical examination, revealing the presence of a normal uterus and cervix, normal ovaries and fallopian tubes, and a large hematocolpos.

**Case presentation:**

This case reports Winter syndrome in a 14-year-old girl which vaginal atresia was managed by a trans perineal vaginal pull through.

**Conclusions:**

Winter syndrome is a rare congenital condition whose clinical picture is that of an adolescent girl with primary amenorrhea and cyclic pelvic pain due to vaginal atresia, varying degrees of renal dysgenesis and deafness due to malformation of the ossicles of the middle ear. Diagnosis is based on clinical examination and imaging. Magnetic resonance imaging allows assessing the importance of atresia and thus guiding surgical management. The goals of surgical intervention are to provide relief from pain, ensure normal sexual intercourse and to preserve fertility. A thorough knowledge of embryology, pre-operative imaging with MRI and clinical examination is essential to plan an appropriate surgical management.

## Background

Distal vaginal agenesis has been described in association with a variety of anomalies. Winter et al. described the association of vaginal atresia, renal aplasia or hypoplasia and anomalies of the ossicles of the middle ear in women with normal development of secondary sexual characteristics and a 46, XX karyotype [1]. Radiographic features may show a hydrometrocolpos or hematometrocolpos due to obstruction [2]. The proper diagnosis and management of this pathology demands a rigorous knowledge of the relevant embryology, anatomy and physiology as well as sensitivity to the psychological effects of the condition.

## Case presentation

We hereby report an uncommon case of a 14-year-old girl, living in a rural area, being first child of two siblings, with a younger brother having no significant pathology, and whose parents are second degree consanguineous. She was born from a full-term pregnancy without significant incident, exposure to radiation or maternal drugs, except for vitamins and iron. Her vaginal delivery in a birth center was without notable incidents, placental examination was normal, but the search for a single umbilical artery was not mentioned.

**Fig. 1:**
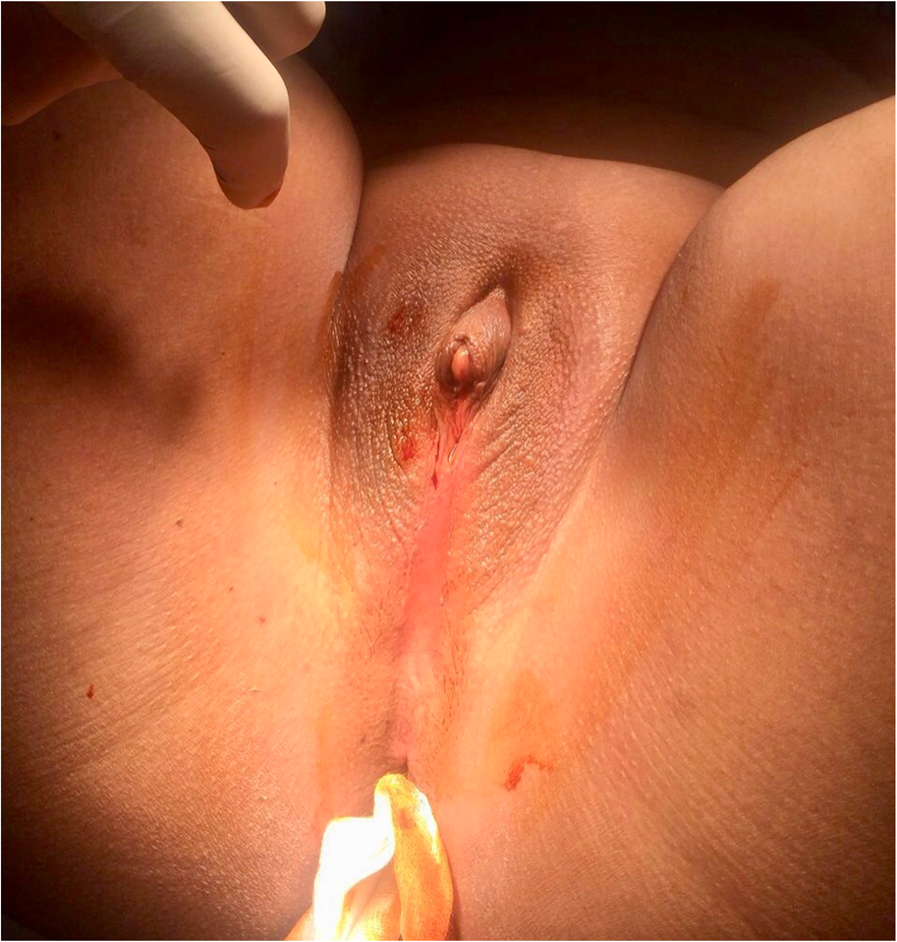
Vulvo-perineal inspection of the clitoral enlargement with hypoplasia of the labia and absence of an external vaginal opening

At 14-year-old, she was admitted at our tertiary care center with complaints of primary amenorrhea and cyclical lower abdominal pain evolving for six months. Clinical examination revealed a normal morphotype, a correct size, well-developed secondary sex characteristics and breast and pubic hair were categorized as Tanner stage 5 and Marshall stade 3 [3]. A mobile nontender mass, arising from the pelvis to the belly button, was felt in the abdomen. Vulvo-perineal inspection revealed clitoral hypertrophy, labia hypoplasia and the absence of an external vaginal opening (Fig. 1). On rectal examination, a tense cystic mass suggestive of hematocolpos was felt anteriorly. An ENT consultation revealed the normal morphology of the pavilions as well as the external auditory canals, however, severe transmission hypoacousia of the right ear with a hearing loss of 70 dB was noted. Abdominal-pelvic ultrasound showed hematocolpos proximal to the perineum and hematometra with an otherwise normal-appearing uterus and ovaries. It also revealed the presence of a small dedifferentiated atrophic right kidney associated with compensatory hypertrophy of the contralateral left kidney. Hormonal assays were performed and within normal range. FSH, LH, and 17b estradiol were normal. Testosterone, D4 androstenedione, 17OH progesterone and DHEA were also normal, which allowed us to eliminate hyperandrogenism. Renal function tests were normal and reflected an effective compensation of the left kidney. The blood karyotype was also normal (46 XX), with no visible chromosomal abnormality.

The diagnosis of poly malformative syndrome associating vaginal, renal and auditory involvement was therefore retained, also called Winter syndrome. A multidisciplinary consultation meeting allowed to opt for a reconstructive surgery of her vaginal agenesis associated with a close monitoring of her auditory and renal functions. Before surgical treatment, the patient and her family were informed about the prognosis of her fertility and verbal as well as written consent were obtained from her legal guardian.

**Fig. 2:**
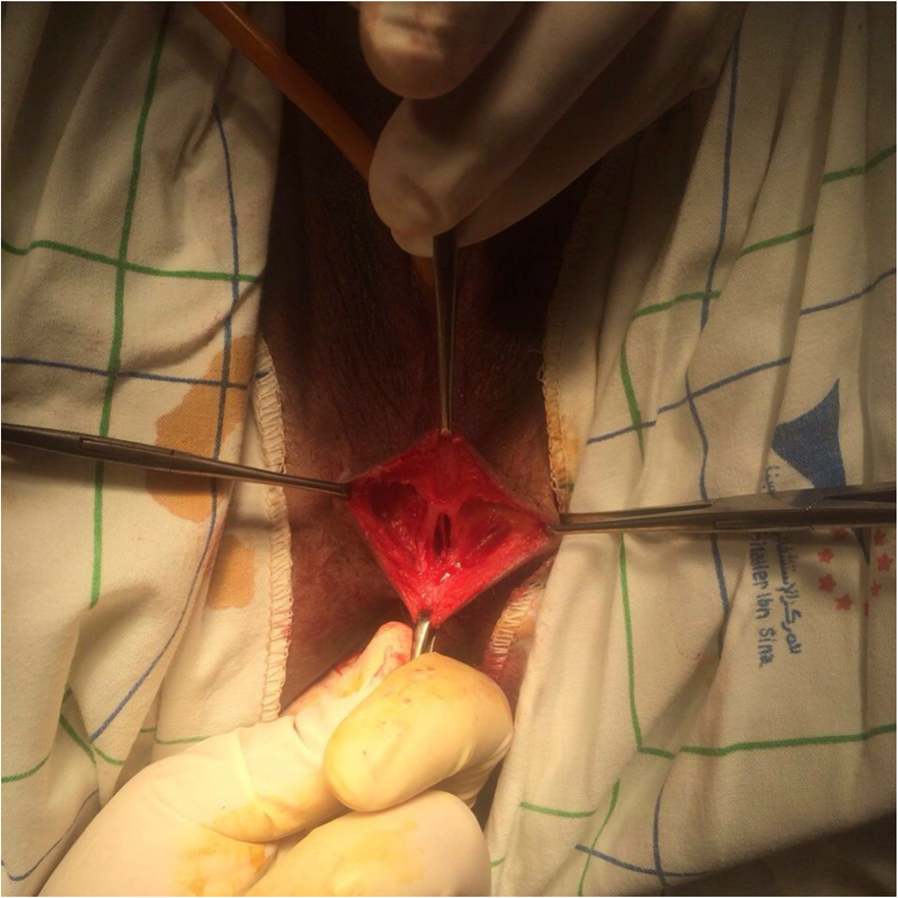
Per operative image of the dissection in the midline to avoid the bladder above and the rectum below

**Fig. 3:**
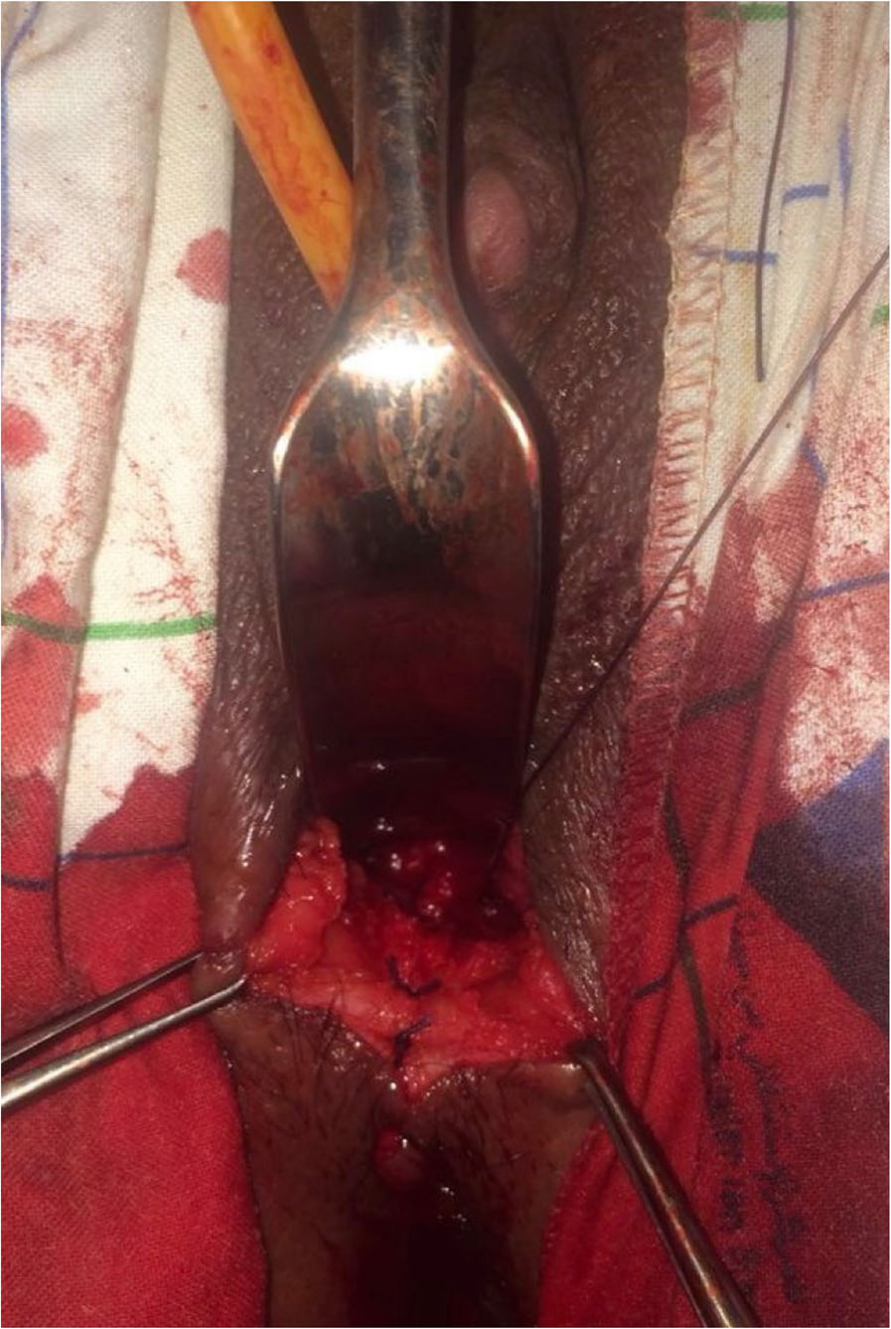
Per-operative image of the neovagina’s radial sutures

**Fig. 4:**
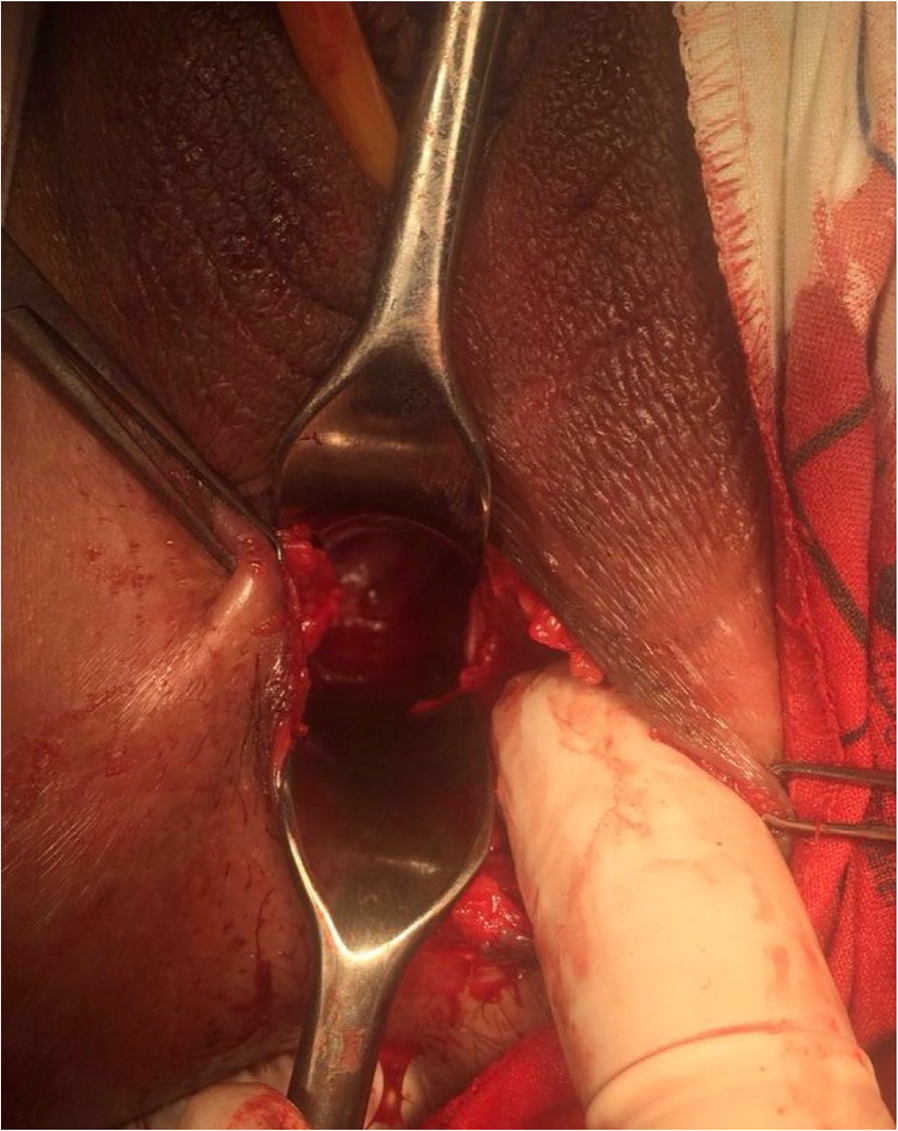
Per-operative image of the neovagina with the cervix external orifice

The vaginoplasty was then carried out with the patient in the dorsal lithotomy position. A Foley catheter is placed into the bladder to avoid an inadvertent anterior entry into the posterior wall of the bladder. A transverse incision is made where the introitus should be located, and a dissection is carried out to reach the obstructed upper vaginal tissue. Care is needed to keep the dissection in the midline and avoid the bladder above and the rectum below (Fig. 2). Creation of a 5 cm-length passage through connective tissue was done carefully to reach the tense bulge of the hematocolpos and allow drainage. Mucosal margins of the upper vagina were then pulled down till the introitus and sutured there with a spacing of 5 mm between each suture, in a radial arrangement (Figs. 3 and 4). The mobilization of the dilated proximal vagina allowed the pull-through vaginoplasty. Operative time was 126 min and blood loss were quantified at 200 ml. Prophylactic antibiotics were initiated postoperatively.

After a successful surgery, we guided the patient on the use of vaginal mold to ensure that the vagina heals well without restenosis. The opening of the vaginal introit was then dilated by ascending vaginal probes of increased dimensions lubricated with estrogen gel, until complete healing and sufficient caliber to sexual intercourse. On follow-up after 2 months, she was regular menstruating with no obstruction signs. At 20-year-old, the young woman became pregnant, and safely vaginally delivered a healthy male baby weighting 2700 g at 38 weeks’ gestation without an episiotomy.

## Discussion and conclusions

Vaginal atresia is a rare congenital anomaly with an incidence ranging from 1 in 4000 to 1 in 10,000 females [4]. It occurs when the urogenital sinus fails to form the distal portion of the vagina, which is replaced with fibrous tissue resulting in utero-vaginal outflow tract obstruction [5]. This genital tract outflow is important for secretion and menstrual effluxion and as a pathway in reproductive function. It may occur as an isolated defect or more commonly associated with various syndromes [6, 7]. Mayer-Rokitansky-Kuster, Kaufman-McKusick, Fraser, Winter and congenital adrenal hyperplasia syndromes are the known examples of such associations [8]. A relatively constant element of these malformations is the association of abnormalities of the genital tract and the urinary system. The embryogenesis of these two systems being intimately linked [9]. In 1941, Gruenwald demonstrated that resection of the Wollfian duct resulted in absent kidney and fallopian tube and unicornuate uterus [10].

Vaginal atresia in Winter syndrome is a distinct condition from vaginal agenesis which is most commonly encountered in women with Rokitansky syndrome (Mayer-Rokitansky-Kuster-Hauser syndrome or Müllerian aplasia) [11]. MRKH syndrome is characterized by a congenital absence of the upper vagina and uterus in a phenotypically, chromosomally and hormonally intact female. While in Winter syndrome, the upper vagina and female reproductive structures are intact, because the differentiation of Mullerian organs is unaffected [8, 12]. Moreover, while Müllerian aplasia confers irreversible sterility, vaginal atresia can be surgically corrected to permit pregnancy [13].

Literature review has allowed us to find five cases of winter syndrome including its first description. In 1968, Winter et al. [1] described a family of four sisters who showed varying degrees of renal dysgenesis, deafness due to malformation of the ossicles of the middle ear, and vaginal atresia [1]. Turner et al’s reported in 1970 a family of four sisters with the eldest one who presented with an abnormal face with a very narrow external auditory meatus with mild deafness associated with absent right kidney and vaginal atresia [14]. The authors suggested that this syndrome might be a recessive disease possibly lethal in the male. McKusick et al’s listed in 1986 the condition as likely autosomal recessive, assigning the number 267400 for his catalog and referenced also Schmidt et al’s report [15–17]. In another family described by King et al. [13] in 1987, one of two affected siblings delivered a normal female infant after vaginal reconstructive surgery. Our patient also delivered a healthy female infant vaginally. Affected siblings and parental consanguinity suggest autosomal recessive inheritance [1, 13]. Franck et al. described in 1982 a small female with clitoral enlargement, hypoplasia of the labia minora, aplasia of the kidney and sensorineural hearing loss [18]. This review of the literature allowed us to note that the Winter syndrome can take on a myriad of presentations. First, renal malformations can lead to renal agenesis as in the case presented by Winter et al. but can also be associated with a bladder hemiatrophy found in the report by Turner et al. [1, 14]. Similarly, auditory malformations mainly concern the middle ear but can also be associated with malformations of the outer ear or even of the face [14]. Growth restriction is also a non-systematic but recurrent symptom, found in 3 out of 5 [1, 14, 18]. Finally, we note the presence of associated rectal malformation found in a single case presented by Turner et al’s [14].. All this leads us to suggest that the Winter syndrome is an entity in its own right, most certainly of autosomal recessive transmission, whose very low recurrence prevents us from fully understanding nor determine the responsible genetic anomaly [17].

Diagnosis can occur at any time; however, most cases are diagnosed in adolescence upon primary amenorrhea and cyclic lower abdominal pain [19]. The clinical picture is that of a girl of pubertal age who consults for primary amenorrhea, whose normo-hormonal character is evident [7]. Anamnesis raises the notion of cyclic chronic pelvic pain [20]. Clinical examination finds a normal morphotype with appropriate Tanner’s staging of secondary sex characteristics [3]. Examination of the perineum reveals absence of vaginal opening [19]. The obstruction can lead to heamatometra, heamatocolpos, endometriosis or pyometra [20]. Trans-abdominal or trans-perineal ultrasound may specify the level of the obstacle [2, 21]. Pelvic ultrasound confirms the diagnosis. It objectifies the image of an hematometra with functional ovaries containing follicles. Transrectal ultrasound can help to analyze the cervix because it provides a precise view of the pelvic organs [22]. Renal ultrasound or intravenous urography will look for an associated urinary malformation [23]. Currently, MRI scanning appears to be the most reliable examination for the diagnosis [24]. It can specify better the height and extent of vaginal aplasia thus making it possible to guide the choice of the most appropriate surgical technique [25].

In our patient, both ovaries with fallopian ducts, uterus, and upper 2/3rd of vagina were normal, therefore, we concluded it to be a urogenital sinus anomaly. She also had a conductive hearing loss and an atrophic right kidney which led us to the diagnosis of Winter syndrome. Therapy is directed to relieve the obstruction of the vaginal outlet and provide normal sexual life and reproductive function [26]. Unlike Rokitansky’s syndrome, vaginoplasty in winter syndrome can allow pregnancy [13]. The management when the atretic segment does not exceed 3 cm is simple. It is done by direct anastomosis of the vaginal mucosa. In contrast, surgery of extended forms is more complex. It uses cutaneous or intestinal grafts [27].

Prophylactic antibiotics initiated postoperatively are important with pull-through vaginoplasty, because the uterus and fallopian tubes may contain blood which is an excellent growth media and because there is a risk of bacteria ascending into what becomes an open system [28]. Postoperatively, the use of dilators ensures that the vagina heals without restenosis [29]. With adequate postoperative dilation, patients will have normal sexual and reproductive function, and vaginal delivery should be possible [30–32]. Our patient was managed successfully by a perineal approach. Symptoms were then relieved, menstrual function was retained and functional vagina was formed.

Winter syndrome is a rare congenital condition whose clinical picture is that of an adolescent girl with primary amenorrhea and cyclic pelvic pain due to vaginal atresia, varying degrees of renal dysgenesis and deafness due to malformation of the ossicles of the middle ear. Diagnosis is based on clinical examination and imaging. Magnetic resonance imaging allows assessing the importance of atresia and thus guiding surgical management. Therapy is directed to relieve the obstruction of the vaginal outlet and provide normal sexual life and reproductive function.

## Data Availability

The datasets used and/or analyzed during the current study are available from the corresponding author on reasonable request.

## Abbreviations

ENT: Ear, Nose, and Throat
MRI: Magnetic Resonance Imaging
MRKH: Mayer-Rokitansky-Kuster-Hauser
